# Endoscopic Decompression of Radiculopathy Caused by Vertebral Artery Loop Formation: Case Report and Literature Review

**DOI:** 10.3390/jcm15103643

**Published:** 2026-05-09

**Authors:** Tae Hoon Yang, In-Suk Bae, Hee In Kang, Jae Hoon Kim, Cheolsu Jwa

**Affiliations:** Department of Neurosurgery, Nowon Eulji Medical Center, Eulji University, Seoul 01830, Republic of Korea; yth@eulji.ac.kr (T.H.Y.); khi2303@eulji.ac.kr (H.I.K.); grimi2@eulji.ac.kr (J.H.K.); chsjwa@daum.net (C.J.)

**Keywords:** vertebral artery loop, cervical radiculopathy, endoscopic spine surgery

## Abstract

**Background:** Cervical radiculopathy due to vertebral artery loop formation (VALF) is rare. This case demonstrates endoscopic posterior foraminotomy after failed conservative treatment. **Methods:** We report a case of VALF treated by means of uniportal full-endoscopic posterior foraminotomy. A focused narrative literature review identified prior surgical cases of VALF-related cervical radiculopathy. **Case description:** A 69-year-old woman had a 4-month right C5 radiculopathy (neck pain, arm radiation, Spurling-positive) due to VALF at C4-5, confirmed via MRI and CT angiography. After failed conservative treatment, full-endoscopic posterior foraminotomy was performed; the symptoms resolved at 3 months. **Conclusions:** Clinicians should be aware that vertebral artery loop formation, although rare, is an important potential cause of cervical radiculopathy. In suspected cases, the vertebral artery should be carefully evaluated with MR or CT angiography to confirm the presence of a loop formation. Full-endoscopic posterior foraminotomy may be technically feasible for carefully selected patients with VALF-related cervical radiculopathy, demonstrating short-term symptom improvement in this case.

## 1. Introduction

Cervical radiculopathy caused by foraminal pathology, either from disc herniation or degenerative narrowing of the foramen, is a very common condition. However, in cases of unresolved radiating pain, clinicians must keep in mind some rare entities, such as cysts, tumors, and vascular malformations, including vertebral artery loop formation (VALF) [1,2,3,4,5].

Whereas conservative therapy is effective in many patients, persistent symptoms may ultimately require surgical intervention. The reported surgical options for VALF include microvascular decompression, foraminotomy with sectioning of the compressed rootlet, and vascular reconstruction; VALF remains an uncommon entity without a standardized treatment algorithm, and various surgical techniques have been described. While posterior decompression for VALF has been described, uniportal full-endoscopic posterior foraminotomy without arterial transposition represents a novel minimally invasive approach.

Herein, we report a case demonstrating the technical feasibility of full-endoscopic posterior decompression for VALF-related radiculopathy, while recognizing the limitations inherent to a single-case experience.

## 2. Focused Narrative Literature Review of Surgically Treated Symptomatic VALF

We conducted a focused narrative review of English-language case reports (1970–2025) describing the surgical treatment of symptomatic VALF (including cervical radiculopathy and related compressive syndromes). Inclusion was based on confirmed neural compression by VALF requiring surgery. Search terms included “cervical”, “radiculopathy”, “vertebral artery”, “loop”, “operation”, and “surgery”, cross-referenced with Boolean operators.

Eligibility was assessed by title and abstract review. Studies were included if they reported surgically treated symptomatic VALF causing cervical radiculopathy or related compressive neurological symptoms. The exclusion criteria comprised trauma to the skull/spine, infections, tumors, or autoimmune diseases. From eligible studies, we extracted the demographics, symptoms, affected level, surgical approach, and outcomes for each patient. In total, 21 studies encompassing 23 patients met the inclusion criteria (Table 1). Because the available evidence consisted almost entirely of isolated case reports and small case series, no formal quality assessment or meta-analytic synthesis was performed.

## 3. Case Description

A 69-year-old woman presented to the neurosurgical department with a 4-month history of neck pain, right upper extremity radiating pain, and a tingling sensation in her arm. Her symptoms were aggravated by the Spurling maneuver and partially relieved upon shoulder abduction. The symptom was prevalent, concordant with right C5 dermatomal innervation. Motor examination showed Medical Research Council grade 5 strength in all extremities, including the deltoid, biceps, wrist extensors, triceps, and hand grip. Sensory examination demonstrated subjective dysesthesia over the right C5 dermatome without a clear distal peripheral nerve distribution. Deep tendon reflexes were symmetric, and no pathologic reflexes were identified; Hoffmann sign and Babinski sign were negative. The differential diagnosis included degenerative cervical foraminal stenosis, shoulder pathology, peripheral nerve entrapment, and brachial plexopathy. Shoulder pathology was considered less likely because the pain was provoked by cervical Spurling maneuver, partially relieved by shoulder abduction, and accompanied by imaging evidence of focal neural compression within the right C4-5 foramen. Peripheral nerve entrapment and brachial plexopathy were considered less likely because the symptoms followed a dermatomal rather than peripheral nerve distribution, and electromyography supported right cervical radiculopathy. Concomitant degenerative cervical disease at the involved level was evaluated on MRI and CT, but no compressive disc herniation or foraminal stenosis sufficient to explain the symptoms was identified apart from the vascular lesion.

**Figure 1:**
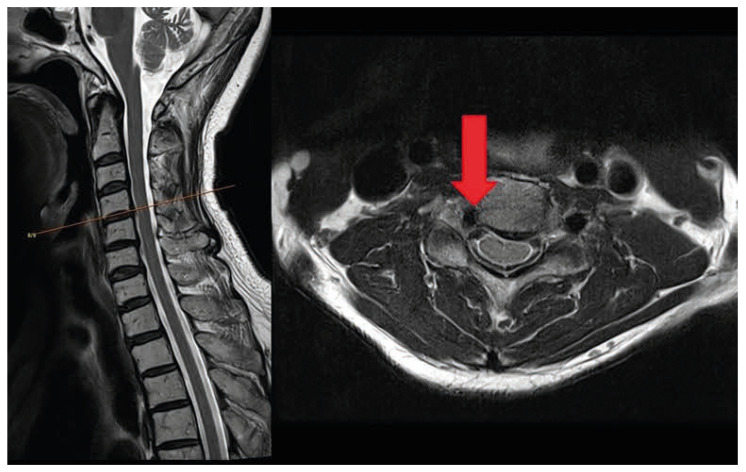
Magnetic resonance imaging of the cervical spine showing a vascular structure occupying the right C4-5 neural foramen (Red arrow).

**Figure 2:**
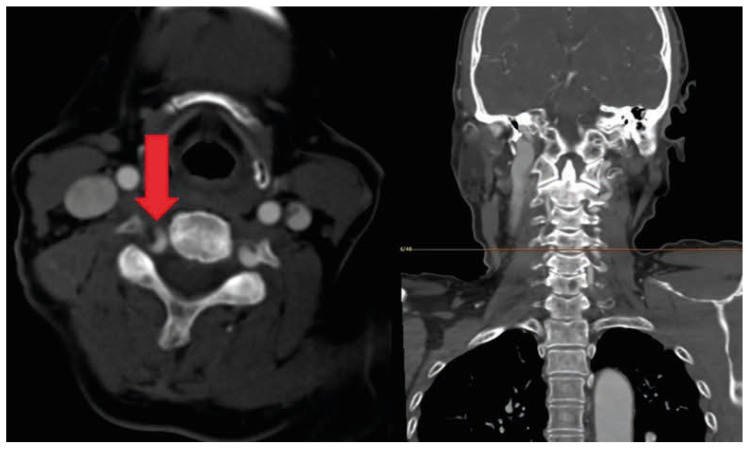
Neck computed tomography angiography demonstrating the vertebral artery coursing into and compressing the right C4-5 neural foramen (Red arrow).

**Figure 3:**
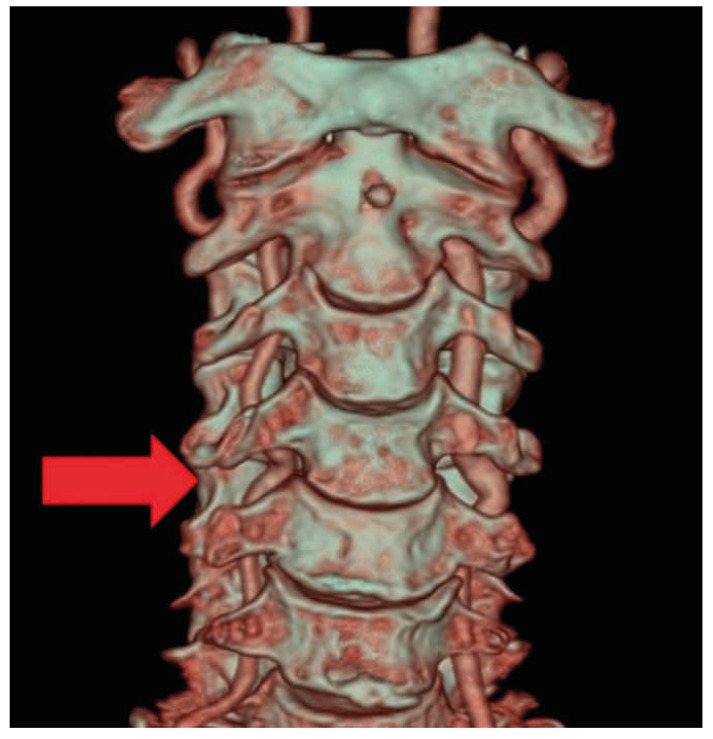
Three-dimensional reconstruction of computed tomography angiography showing a vertebral artery loop at the right C4-5 level (Red arrow).

Magnetic resonance imaging (MRI) of the cervical spine revealed a vascular structure in the right C4-5 foramen compressing the right C5 nerve root (Figure 1). Neck computed tomography angiography (CTA) also revealed a vertebral artery in the right C4-5 foramen (Figure 2). Three-dimensional reconstruction produced an image consistent with vertebral artery loop formation at the right C4-5 level (Figure 3). Electromyography (EMG) confirmed right cervical 5th radiculopathy. Conservative management was attempted for approximately 3 months and included non-steroidal anti-inflammatory drugs, pregabalin, and physical therapy. These measures provided only limited and temporary symptom relief, and the patient continued to experience persistent radicular pain that interfered with daily activities. Because the symptoms remained refractory despite adequate nonoperative treatment and imaging demonstrated a structurally compressive vascular lesion at the symptomatic level, surgical treatment was considered. Cervical transforaminal or selective nerve root injection was not pursued because of the vascular nature of the lesion and the potential risk of vertebral artery injury.

**Figure 4:**
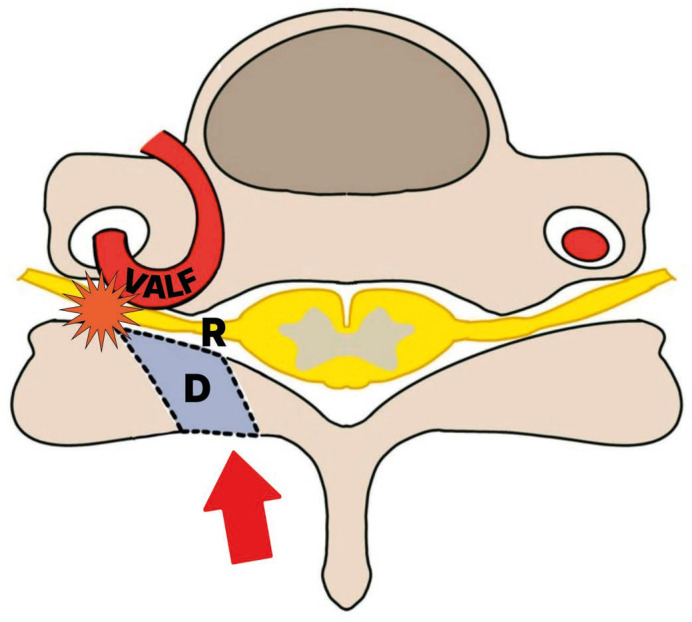
Illustration showing C5 nerve root compression by VALF and endoscopic posterior decompression corridor (red arrow). VALF, vertebral artery loop formation; R, root; D, decompression part.

**Figure 5:**
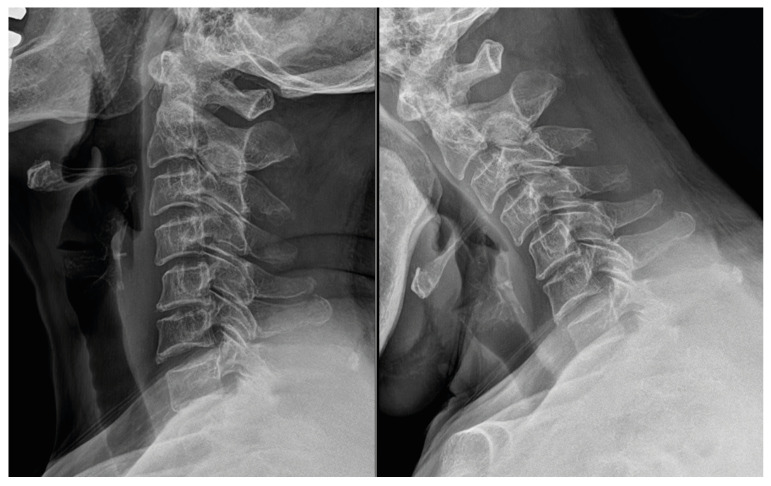
Postoperative plain radiography demonstrating no instability after endoscopic posterior decompression.

After 3 months of failed conservative management, uniportal full-endoscopic posterior C4-5 foraminotomy was performed in the prone position under general anesthesia. The V-point (medial facet border) was identified fluoroscopically. Intraoperative navigation, Doppler ultrasonography, and neuromonitoring were not used in this case. To minimize vascular risk, surgical exposure was deliberately limited to the minimum extent necessary for neural decompression, and complete circumferential dissection of the vertebral artery loop was intentionally avoided. In preparation for possible vertebral artery injury, hemostatic agents and microsurgical instruments were prepared in advance, and conversion to an open microscopic surgery with tamponade and vascular control was planned as a bailout strategy if uncontrolled arterial bleeding occurred. Sequential dilators created a working channel; drilling targeted the superior lamina inferior edge to the medial inferior/superior articular processes. Approximately 20% of the facet joint was resected. Ligamentum flavum was resected, exposing dura and venous plexus. Radiofrequency ablation achieved hemostasis. A nerve hook was then used to gently dissect the C5 root away from the vascular loop, which was located ventral to the nerve root, under fluoroscopic guidance. Complete circumferential exposure of the vertebral artery loop was not attempted; instead, decompression was judged by adequate mobilization of the nerve root and relief of ventral compression. No intraoperative video was recorded, given the retrospective nature of this single-case report (Figure 4 illustrates the decompression). This point is emphasized as a technical limitation of the procedure. An anti-adhesion agent (X-Block^®^, BMI Korea, Seoul, Republic of Korea) was applied. The operative time was 50 min, and the estimated blood loss was 20 mL. Extension and flexion X-ray imaging were performed 2 months after surgery, and no abnormalities such as instability were confirmed (Figure 5). At the 3-month follow-up, symptoms had resolved completely (VAS-neck 0/10, VAS-arm 0/10), and no complications were observed. Nevertheless, this follow-up duration is insufficient to draw conclusions regarding long-term durability, recurrence, or delayed instability.

## 4. Discussion

VALF represents a tortuous course of the VA that may protrude into the neural foramen or spinal canal. The clinical significance of VALF is twofold: it can directly compress neural structures, resulting in radiculopathy, and it markedly increases the risk of catastrophic vertebral artery injury during cervical spine surgery or transforaminal epidural steroid injections [17]. Clinicians should therefore recognize VALF as both a potential pain generator and an important vascular hazard in the cervical spine.

The pathophysiology of VALF-related radiculopathy primarily involves mechanical compression of the cervical nerve root by the aberrant arterial loop [17]. Dynamic factors may also contribute, as arterial pulsation and cervical motion can exacerbate irritation of the nerve root, which is consistent with the reproduction of radicular symptoms on provocative maneuvers such as the Spurling test [22]. This mechanism explains how a vascular lesion can mimic a typical degenerative cervical lesion both clinically and radiologically.

Few surgically treated cases of symptomatic VALF causing cervical radiculopathy or other neural compressive symptoms have been reported in the literature. The radiographic prevalence of VALF is 0.6–7.5%; symptomatic radiculopathy is much rarer [1,2,3,4,5]. Once VALF-related radiculopathy is identified, conservative management is appropriate initially, while surgery may be considered for persistent symptoms or neurological deficits despite adequate nonoperative treatment. Surgical treatment should therefore be considered in patients with refractory pain or neurological deficits despite adequate conservative management. Various surgical strategies have been described, including anterior and posterior decompression, microvascular decompression (MVD), vascular reconstruction and, in selected cases, endovascular coiling [7,11,14,15,25].

Our review of the literature identified 21 articles including 23 patients who underwent surgical management for symptomatic VALF causing cervical radiculopathy or related neural compressive symptoms between 1970 and 2025 (Table 1). Among these, 12 patients were treated via an anterior approach for bony decompression with or without MVD, 9 via posterior decompression, 1 with endovascular coiling, and 1 with anterior cervical discectomy and fusion. In cases using MVD, surgeons employed either an interposition graft (such as Teflon or Dacron) or an allograft sling to transpose or separate the vertebral artery loop from the affected nerve root.

Surgically, the anterolateral approach permits the direct exposure and relocation or reconstruction of the vertebral artery loop, thereby addressing the primary compressive pathology. This route offers the possibility of arterial transposition or re-anastomosis but carries potential risks such as injury to the recurrent laryngeal nerve, dysphagia, and direct vascular damage, even though these complications have rarely been reported in published series [19]. In contrast, the posterior approach is more familiar to most neurosurgeons and provides direct access to the exiting nerve root, allowing direct decompression of the neural elements and indirect decompression of the artery without necessarily manipulating the vertebral artery loop itself.

However, the posterior approach has important limitations. It often requires more extensive bone removal, including laminectomy and foraminotomy, and may necessitate drilling of the transverse foramen to mobilize the artery. Because the cervical nerve root typically lies between the vertebral artery and the surgeon, visualization and control of the artery can be limited, increasing the risk of nerve injury and iatrogenic instability that may require additional fusion [19]. Thus, the choice of approach should be individualized, balancing the need for definitive decompression against the risks of vascular and neural complications.

Although the anterior approach is more frequently reported, several authors have shown that a posterior approach for vertebral artery loop-related nerve compression is also safe and effective, with good rates of symptomatic improvement. In previously described posterior cases, surgeons performed direct decompression of both the nerve root and the vertebral artery using combinations of foraminotomy, partial facetectomy, and MVD. Nevertheless, both anterior and posterior open procedures carry an inherent risk of vertebral artery injury and potential subsequent posterior circulation infarction.

In our case, we treated the patient using a uniportal full-endoscopic posterior approach through an approximately 1-inch incision. Although the vertebral artery loop could not be completely visualized, the compressed cervical nerve root was sufficiently decompressed from the dorsal aspect, and careful dissection of the ventral portion of the root using a hook created a safe corridor between the VALF and the nerve. The use of an endoscopic drill, combined with frequent palpation of the medial pedicle wall using a nerve hook, helped to limit facet joint resection and may reduce the risk of iatrogenic segmental instability compared with more extensive open posterior decompression. However, this surgical approach involves trade-offs, including limited visualization of the arterial loop, and no single approach can currently be considered standard for this rare pathology.

This experience suggests that radiculopathy caused by vascular malformations such as VALF can be effectively managed by selectively decompressing the posterior aspect of the compressed nerve root using minimally invasive full-endoscopic surgery in carefully selected patients. Rather than focusing on detailed technical nuances, our report emphasizes the conceptual feasibility of treating VALF-related cervical radiculopathy with an endoscopic posterior approach that avoids direct manipulation of the vertebral artery. Unlike prior posterior cases requiring MVD or extensive facetectomy, our approach used full-endoscopic decompression without direct arterial manipulation. As endoscopic spine techniques continue to evolve, this strategy may represent an attractive alternative to traditional open procedures in carefully selected patients.

Clinicians should maintain a high index of suspicion for vertebral artery loop formation in patients with atypical or refractory cervical radiculopathy, especially when imaging reveals unusual foraminal vascular structures or bony erosion. In suspected cases, preoperative MR angiography or CT angiography is essential to confirm the presence and exact course of the loop and to guide surgical planning while minimizing the risk of iatrogenic vascular injury. Full-endoscopic posterior decompression, as demonstrated in this case, may offer a minimally invasive option after failure of conservative management; however, broader claims regarding safety and efficacy require additional clinical experience and long-term follow-up.

## 5. Conclusions

This report describes a rare case of vertebral artery loop formation (VALF) causing refractory cervical radiculopathy, which was treated with uniportal full-endoscopic posterior foraminotomy. This case demonstrates the technical feasibility of uniportal full-endoscopic posterior foraminotomy for VALF-related cervical radiculopathy in a carefully selected patient, achieving short-term symptom relief without arterial manipulation. Nevertheless, conclusions regarding reproducibility, long-term outcomes, and overall safety should remain cautious.

## Figures and Tables

**Table 1:** Reported surgical cases of symptomatic vertebral artery loop formation (VLAS) causing cervical radiculopathy or related neural compressive symptoms.

Authors, Year	Sex, Age	VALF-Related Symptoms	Level	Surgical Technique	Outcome
Zimmerman et al., 1970 [6]	F, 50	Cervical pain, Lt. occipital pain	C4-5, Lt.	Posterior decompression	Asymptomatic at 10 months
Anderson et al., 1970 [7]	F, 54	Facial pain, Lt. neck pain	C3-4, Lt.	Posterior decompression	Asymptomatic for several months
Sharma et al., 1993 [8]	F, 75	Occipital neuralgia, cervical myelopathy	C2-3, Lt.	Posterior decompression and fusion, MVD with Surgicel	Residual spasticity at 1.5 years after surgery
Satoh et al., 1993 [9]	F, 59	Lt neck and arm pain	C1, Lt	Suboccipital decompression	Asymptomatic at 2 years
Duthel et al., 1994 [10]	F, 37	Lt arm pain, shoulder pain	C5-6, Lt	Anterolateral decompression, MVD with Teflon	Asymptomatic at 3 months
Detwiler et al., 1998 [11]	M, 70	Rt neck pain	C3-4, Rt	Posterior decompression with MVD	Asymptomatic at 2 years
Sakaida et al., 2001 [12]	M, 62	Lt shoulder pain, arm pain	C4-5, Lt	Anterolateral decompression, VA transection with anastomosis	Asymptomatic at 2 years
Korinth et al., 2007 [13]	F, 68	Cervical radiculopathy	C4-5, Rt	Anterolateral decompression, MVD with Teflon	Follow-up length not reported
Dahdaleh et al., 2010 [14]	M, 55	Neck pain, shoulder pain	C2-3, C3-4, Lt.	Post-cervical fusion w/o decompression	Asymptomatic at 6 months
Hage et al., 2012 [15]	F, 27	Cervical radiculopathy	C6-7, Rt	Anterolateral decompression, MVD	Asymptomatic at 13 months
Chibbaro et al., 2012 [2]	F, 50	Cervical radiculopathy	C5-6, Lt	Anterolateral decompression, MVD	Asymptomatic at 1 year
Tandon et al., 2013 [16]	F, 52	Neck pain, arm pain	C4-5, Rt	Anterolateral decompression, MVD with sling	Asymptomatic at 1 year
Ekşi at al., 2016 [17]	M, 60	Neck pain, Lt arm weakness	C5-6, Lt.	Posterior decompression	Follow-up length not reported
Ju et al., 2017 [18]	F, 52	Neck pain, Lt arm numbness	C6-7, Lt	Anterolateral decompression, MVD with sling	Follow-up length not reported
Wang et al., 2017 [19]	F, 51	Cervical radiculopathy	C5-6, Lt	Anterolateral decompression, MVD with Teflon	Numbness at 4 months
Wang et al., 2017 [19]	F, 49	Neck pain, occiput pain	C3-4, Lt.	Anterolateral decompression, MVD	Improved at 6 months
Venteicher et al., 2019 [20]	F, 72	Cervical radiculopathy	C4-5, Rt	Anterolateral decompression, MVD with pledget	Asymptomatic at 1 year
Khansuheb et al., 2020 [21]	F, 62	Cervical radiculopathy	C6-7, Lt	Endovascular coiling for VA sacrifice	Asymptomatic at 9 months
Wood et al., 2021 [4]	M, 35	Cervical radiculopathy	C5-6, Lt.	Anterolateral decompression, MVD with Dacron	Follow-up length not reported
Wood et al., 2021 [4]	F, 48	Cervical radiculopathy	C3-4, C4-5, Lt	Anterolateral decompression, MVD with Dacron	Follow-up length not reported
Farshad et al., 2022 [22]	F, 76	Cervical radiculopathy	C5-6, Rt	Anterior discectomy and fusion, foraminotomy	Asymptomatic at 1 year
Semonche et al., 2024 [23]	M, 49	Neck pain, migraine	C3-4, Lt	Posterior decompression and fusion, MVD with Teflon	Asymptomatic at 1 year
Benato et al., 2025 [24]	M, 57	Neck pain, shoulder pain	C5-6, Lt.	Posterior decompression, MVD with grafton	Follow-up length not reported

MVD, microvascular decompression; VA, vertebral artery.

## Data Availability

The data presented in this study are available upon request from the corresponding author. No new data were created or analyzed in this study.

## Abbreviations

The following abbreviations are used in this manuscript:

VALF	Vertebral artery loop formation
VA	Vertebral artery
MRI	Magnetic resonance imaging
CTA	Computed tomography angiography
VAS	Visual analogue scale
MVD	Microvascular decompression
